# Primary Leiomyosarcoma of the Breast in a Patient With a History of Phyllodes Tumor

**DOI:** 10.7759/cureus.106439

**Published:** 2026-04-04

**Authors:** Krishna Athreya Thutupalli, Arulappan Thangasamy, Sivaraja Parthipan Kasithangam

**Affiliations:** 1 General Surgery, Sri Ramachandra Institute of Higher Education and Research, Chennai, IND

**Keywords:** breast disease, breast leiomyosarcoma, breast parenchymal disease, soft-tissue sarcoma, wide local excision (wle)

## Abstract

Primary breast leiomyosarcoma is a rare mesenchymal malignancy, thought to arise from smooth muscle elements of the nipple-areolar complex, vascular structures, or stromal tissue. Owing to its low incidence, current knowledge is largely derived from isolated case reports and small case series. Patients typically present with a painless, gradually enlarging breast mass, while imaging findings are often non-specific, posing diagnostic challenges. Definitive diagnosis relies on histopathological evaluation supplemented by immunohistochemistry (IHC) to differentiate it from other spindle cell neoplasms of the breast. Treatment strategies are extrapolated from the management of soft-tissue sarcomas at other sites, with wide local excision and negative surgical margins forming the mainstay of therapy. The role of adjuvant radiotherapy and chemotherapy remains unclear due to limited evidence and a lack of consensus guidelines. Given its rarity, reporting such cases is essential to enhance understanding of its clinical behavior, optimize management approaches, and inform future therapeutic recommendations.

A patient in her 30s, status post a simple mastectomy two years ago (histopathological diagnosis: borderline malignant phyllodes tumor), presented with a rapidly growing right chest wall swelling. Ultrasound of the swelling showed an oval, well-circumscribed, heterogeneous lesion measuring 3.9 cm × 2.8 cm, with solid and cystic components in the right chest wall. Fine-needle aspiration showed clusters of spindle cells with nuclear atypia. Wide local excision demonstrated a high-grade spindle cell tumor, confirmed by histopathology and IHC (smooth muscle actin positive, Ki-67 >10%) as leiomyosarcoma. On follow-up, no evidence of residual disease or distant metastasis was identified. This case highlights the challenges in diagnosing rare breast sarcomas, the potential for recurrence or transformation of phyllodes tumors, and the reliance on complete surgical excision followed by adjuvant radiotherapy for optimal local disease control.

## Introduction

Soft-tissue tumors comprise a heterogeneous group of neoplasms arising from mesenchymal tissues such as smooth muscle, adipose tissue, fibrous tissue, peripheral nerves, and vascular structures. These tumors are classified according to their morphological resemblance to normal mesenchymal tissues and their presumed cell of origin. Although the majority of soft-tissue tumors are benign, soft-tissue sarcomas (STS) represent a rare group of malignant mesenchymal neoplasms, accounting for less than 1% of adult malignancies.

STS typically present as a painless, enlarging mass, often discovered incidentally by the patient. Clinical features that raise suspicion for malignancy include a tumor size greater than 5 cm, progressive increase in size, swellings located deep to the deep fascia, the presence of pain, fixation to surrounding structures, and recurrence following previous excision [1].

Evaluation of a suspected soft-tissue sarcoma involves a systematic approach incorporating clinical assessment, imaging, and histopathological confirmation. Magnetic resonance imaging (MRI) is generally the investigation of choice, as it provides detailed delineation of tumor extent, relationship to surrounding structures, and involvement of fascial compartments [2]. Since STS most commonly metastasizes hematogenously to the lungs, staging investigations routinely include chest CT.

Definitive diagnosis is achieved by image-guided core-needle biopsy. Fine-needle aspiration is generally inadequate due to the limited tissue architecture available for histologic characterization [3]. Biopsy planning is critical, as the biopsy tract should subsequently be incorporated into the definitive surgical resection to minimize the risk of tumor seeding and local recurrence.

Surgical excision with adequate margins of normal tissue remains the cornerstone of treatment for localized STS. Adjuvant or neoadjuvant radiotherapy is commonly employed to improve local control, although the optimal timing remains debated [4]. Chemotherapy has a more limited and histology-specific role, with agents such as doxorubicin and ifosfamide used in selected high-risk or metastatic cases. Despite advances in multimodal therapy, tumor grade remains the most important predictor of distant metastasis, with the lungs representing the most frequent site of dissemination.

Among the various histological subtypes of soft-tissue sarcoma, leiomyosarcoma is a malignant tumor arising from smooth muscle cells and may occur in diverse anatomical locations. Primary leiomyosarcoma of the breast is exceptionally rare, accounting for an estimated 0.0006% of all breast cancers, with fewer than 50 cases documented in the literature [5]. Leiomyosarcomas can arise de novo from the smooth muscle cells lining blood vessels within the mammary stroma, or potentially secondary to malignant stromal transformation within pre-existing tumors such as cystosarcoma phyllodes [6,7]. Owing to its rarity, the clinical characteristics, optimal management strategies, and long-term outcomes of this entity remain incompletely defined. The present report describes a case of primary breast leiomyosarcoma and reviews the relevant literature.

## Case presentation

**Figure 1:**
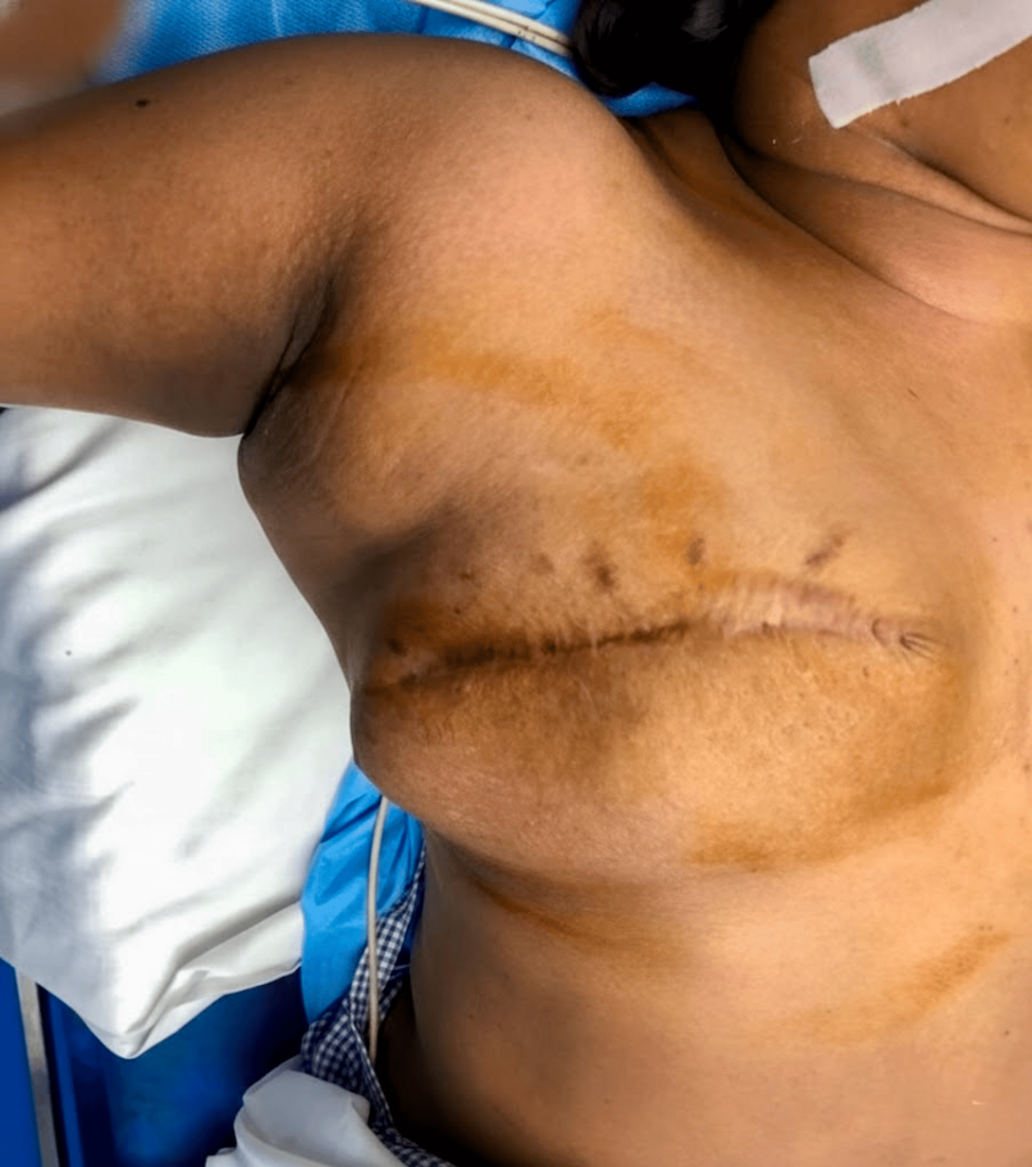
Preoperative image showing right chest wall tumor

A woman in her early 30s presented with a painless, rapidly enlarging lump in the right chest wall for two months. Her past surgical history included a right simple mastectomy performed two years earlier at a different hospital for a phyllodes tumor of borderline malignant potential. The patient had no other significant medical, social, or family history relevant to her current presentation. On clinical examination, a well-healed transverse surgical scar was noted on the right anterior chest wall. Inferior to this scar, a solitary, firm, nodular swelling measuring approximately 5 cm × 4 cm, with well-defined borders, was palpated (Figure 1).

The mass was mobile relative to the deep tissues. There was no palpable axillary lymphadenopathy.

Investigations

Ultrasound of Bilateral Breasts

**Figure 2:**
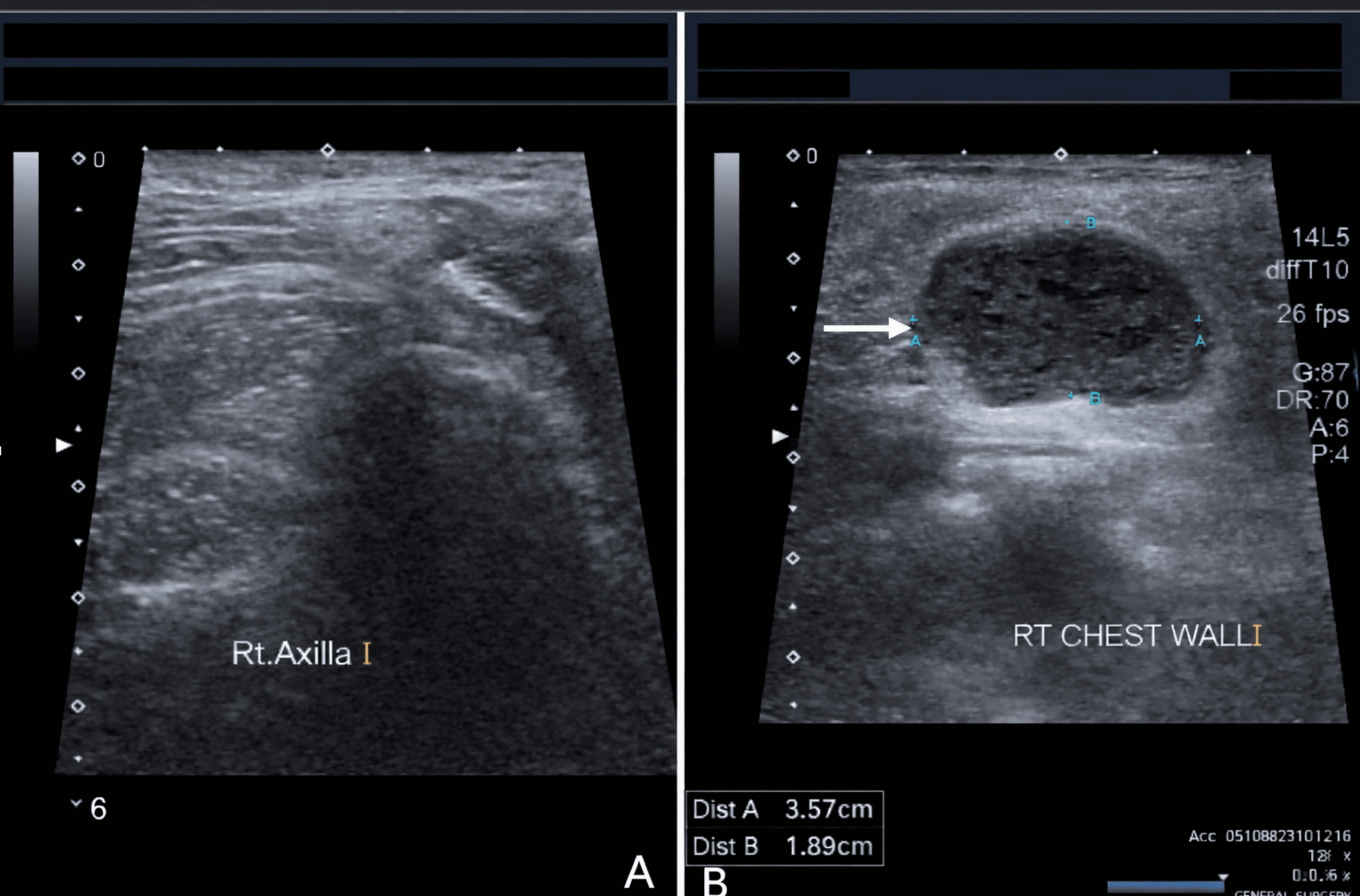
Ultrasonogram images of the right axilla (A), showing a clear axilla with no lymph nodes, and the right chest wall tumor (B), indicated by a white arrow, demonstrating well-defined borders without areas of calcification, measuring 3.57 cm × 1.86 cm

**Figure 3:**
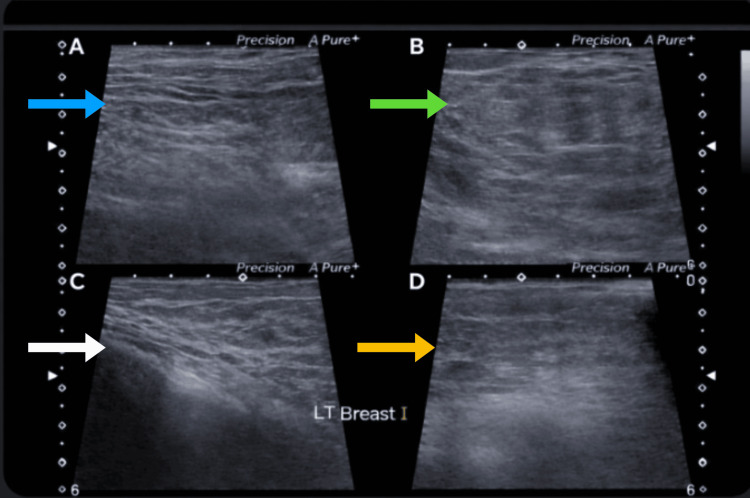
Ultrasonogram of the left breast showing the upper inner quadrant (A, blue arrow), upper outer quadrant (B, green arrow), lower inner quadrant (C, white arrow), and lower outer quadrant (D, yellow arrow), demonstrating predominantly heterogeneous tissue composition with no solid or cystic lesions, a normal nipple-areolar complex, no ductal dilatation, and a normal retroareolar region

An oval, well-circumscribed, heterogeneous lesion measuring 3.9 cm × 2.8 cm was identified within the residual right chest wall. The mass contained both solid and cystic areas (Figures 2, 3).

Fine Needle Aspiration Cytology

Clusters of malignant spindle cells mixed with epithelial cells were observed. Several cells exhibited nuclear atypia in a background of hemorrhage, raising suspicion for malignancy, likely a sarcoma.

Differential diagnoses

Local recurrence of borderline phyllodes tumor was the most immediate concern given the patient’s history. Recurrent phyllodes tumors, especially those previously classified as borderline, can show increased stromal cellularity and atypia. Malignant phyllodes tumor, representing a high-grade recurrence of the stromal component, would also be characterized by a spindle cell population. A new primary soft-tissue sarcoma (leiomyosarcoma or fibrosarcoma) may arise de novo from the mammary stroma or chest wall.

The definitive diagnosis was indicated by the high-grade spindle cell morphology and the immunohistochemistry (IHC) results. The strong positivity for smooth muscle actin (SMA) confirmed the smooth muscle differentiation, establishing the final diagnosis as leiomyosarcoma and effectively ruling out other spindle cell malignancies like fibrosarcoma or metaplastic carcinoma.

Treatment

The patient underwent wide local excision of the recurrent right chest wall tumor. Intraoperatively, residual breast parenchyma was noted adjacent to the tumor. The patient underwent a wide local excision of the swelling in the right chest wall.

**Figure 4:**
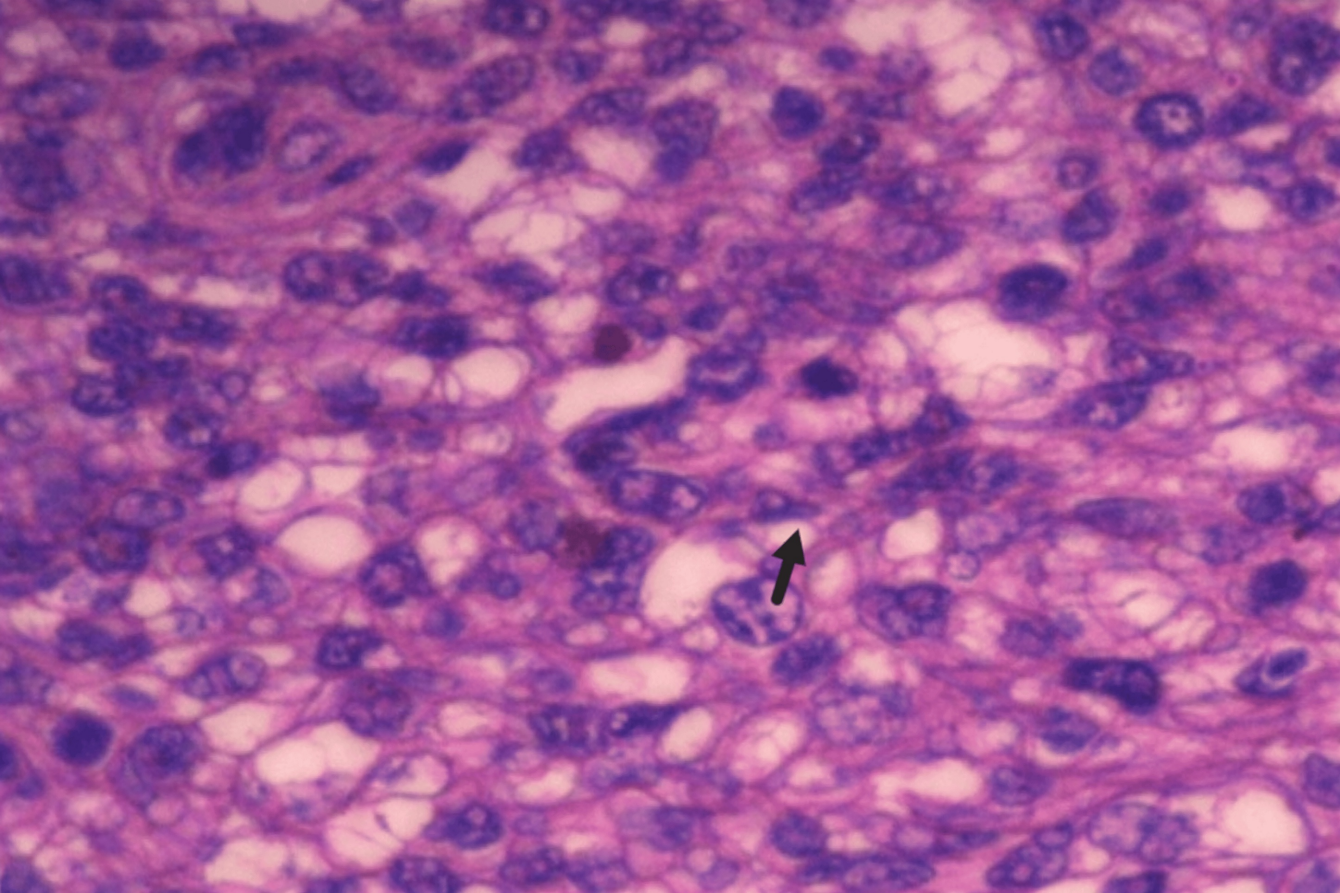
Microscopic image of the specimen showing a hypercellular lesion with atypical stromal cells demonstrating moderate nuclear pleomorphism (arrow) and areas of myxoid differentiation

**Figure 5:**
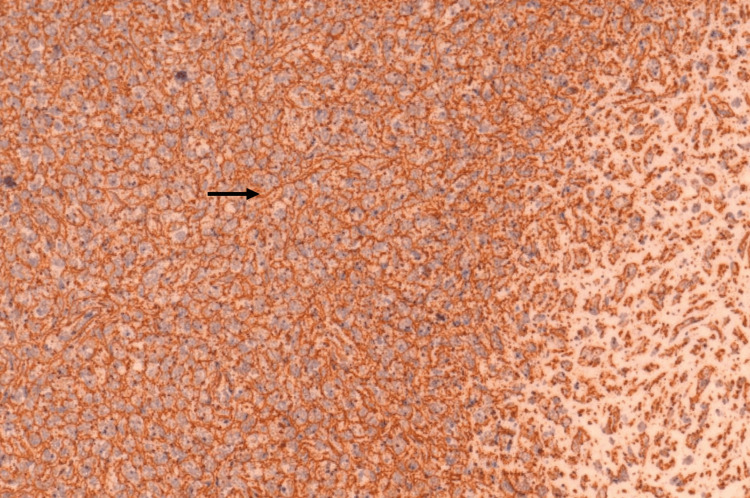
IHC showing SMA staining (arrow), indicating leiomyosarcoma IHC: immunohistochemistry; SMA: smooth muscle actin

**Figure 6:**
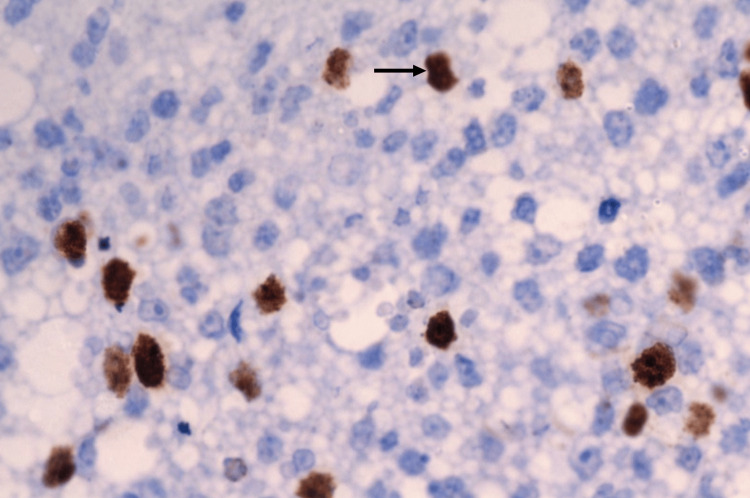
Ki-67 staining positive (arrow), indicating a higher proliferative rate and aggressive tumor behavior

Histopathology (HPE) and IHC revealed a hypercellular lesion showing atypical stromal cells with moderate nuclear pleomorphism and areas showing myxoid differentiation (Figure 4). These cells displayed a high nuclear-to-cytoplasmic ratio and increased mitotic activity. IHC showed positivity for SMA (Figure 5) and a high Ki-67 index (>10%) (Figure 6). This profile confirmed the diagnosis of a high-grade leiomyosarcoma, likely arising in the residual breast parenchyma or as a malignant transformation of the stromal component of the previous phyllodes tumor. The margins of the resected specimen were microscopically free of tumor.

The post-operative period was uneventful, and the patient was discharged on the second post-operative day.

Follow-up

**Figure 7:**
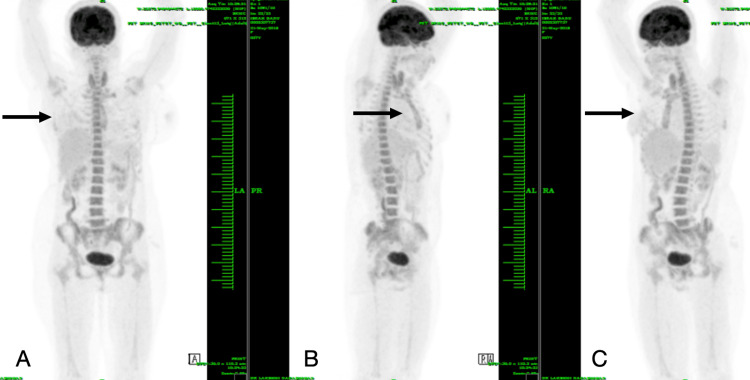
Postoperative PET-CT image showing no uptake in the region of the tumor. (A) Coronal view; (B) sagittal view; (C) oblique view. Arrows indicate the preoperative location of the tumor in the different views PET-CT: positron emission tomography-computed tomography

Postoperatively, a positron emission tomography-computed tomography (PET-CT) scan was performed to stage the disease and assess for residual tumor. The scan confirmed the absence of residual disease, locoregional lymphadenopathy, or distant metastasis (Figure 7). Given the high-grade nature of the leiomyosarcoma and its recurrence, the patient was planned for adjuvant radiotherapy (25 fractions) to the right chest wall to reduce the risk of local recurrence. The patient is on regular follow-up. At three months post-surgery, the patient is healthy and has returned to her normal daily activities while completing the planned radiotherapy regimen.

## Discussion

STS are a heterogeneous group of malignancies that, while commonly arising in the extremities and trunk, are exceedingly rare in the breast [8]. Among STS, leiomyosarcoma represents one of the more frequent histological subtypes, typically occurring in the sixth and seventh decades of life; hence, its occurrence in a younger patient is unusual. Although risk factors such as prior radiation exposure have been implicated in the pathogenesis of leiomyosarcoma, no such predisposing factor was identified in the present case. Radiation-induced sarcomas are a recognized but rare entity, typically developing after a latency period of several years following radiotherapy, most commonly in the setting of breast irradiation; however, leiomyosarcoma is a less frequent subtype compared to angiosarcoma in this context [9].

The rarity of primary breast leiomyosarcoma contributes to its diagnostic challenge, as both clinical and radiological features are often non-specific. Definitive diagnosis relies on histopathological examination supplemented by IHC [10]. Characteristically, tumor cells demonstrate positivity for smooth muscle markers such as SMA and desmin, while lacking expression of epithelial markers (cytokeratins) and melanocytic markers (S-100). In patients with a prior history of phyllodes tumor, the possibility of sarcomatous transformation must be considered, particularly in recurrent spindle cell lesions [11].

Surgical excision with negative margins remains the cornerstone of treatment for localized disease. Achieving adequate surgical margins is critical, as margin status is one of the most important predictors of local recurrence [2]. While a margin of at least 1 cm has traditionally been advocated, contemporary sarcoma management emphasizes complete excision with histologically negative margins (R0 resection), even if narrower, provided critical structures are preserved [2]. In cases of close or positive margins, re-excision should be considered where feasible.

Unlike epithelial breast malignancies, lymphatic spread is uncommon in sarcomas, with hematogenous dissemination, most frequently to the lungs, being the predominant pattern of metastasis [2,12]. Consequently, routine axillary lymph node dissection or sentinel lymph node biopsy is not indicated unless there is clinically or radiologically suspicious nodal involvement [13]. Local recurrence is primarily related to inadequate surgical margins, whereas distant metastasis is more closely associated with tumor grade and size [2,12].

The role of radiotherapy in the management of breast leiomyosarcoma is extrapolated from data on STS at other anatomical sites. Adjuvant radiotherapy has been shown to improve local control, particularly in high-grade tumors, lesions larger than 5 cm, or when surgical margins are close or positive [2,14]. Neoadjuvant radiotherapy offers theoretical advantages, including tumor downstaging, improved resectability, and smaller radiation fields; however, it is associated with higher rates of wound complications [15]. In contrast, adjuvant radiotherapy is more commonly employed in breast sarcomas due to improved wound healing, although it may involve larger treatment volumes [15]. Given the absence of randomized trials specific to breast leiomyosarcoma, the decision between neoadjuvant and adjuvant radiotherapy should be individualized based on tumor characteristics and multidisciplinary team discussion.

The role of systemic chemotherapy in leiomyosarcoma remains limited and is generally reserved for high-risk, metastatic, or unresectable disease. Anthracycline-based regimens, particularly doxorubicin with or without ifosfamide, remain the standard first-line therapy, while combinations such as gemcitabine and docetaxel may be used in selected cases. However, the benefit of adjuvant chemotherapy in localized disease remains controversial and has not consistently demonstrated a survival advantage.

Prognosis in leiomyosarcoma is primarily determined by tumor grade, size, and margin status. High-grade tumors and inadequate surgical margins are associated with an increased risk of local recurrence and distant metastasis [2,12]. Long-term surveillance is essential, as recurrences may occur several years after initial treatment, most commonly involving the lungs. Follow-up protocols typically include periodic clinical examination and imaging, particularly chest imaging, to detect metastatic disease at an early stage.

Given the rarity of primary breast leiomyosarcoma, there is a lack of standardized treatment guidelines, and management is largely guided by principles derived from soft-tissue sarcoma care. Continued reporting of such cases is crucial to enhance understanding of tumor behavior, refine treatment strategies, and improve patient outcomes.

## Conclusions

Primary leiomyosarcoma of the breast is an extremely rare, high-grade soft-tissue sarcoma that requires a high index of suspicion in the evaluation of a rapidly growing breast or chest wall mass. The pathological diagnosis is confirmed by HPE examination showing malignant spindle cells and IHC demonstrating smooth muscle differentiation (e.g., strong positivity for SMA). Complete surgical resection with negative margins is the single most important factor for curative intent and local disease control. Adjuvant radiotherapy is strongly recommended for high-grade or recurrent breast sarcomas to reduce the risk of local recurrence, following guidelines for STS. A prior history of borderline phyllodes tumor warrants close follow-up due to the risk of rare, aggressive stromal recurrence or malignant transformation.
